# Supplementary material for: Reprogramming of cis-regulatory networks during skeletal muscle atrophy in male mice
Source: Nat Commun. 2023 Oct 18;14:6581. doi: 10.1038/s41467-023-42313-3 (PMC10584982; doi:10.1038/s41467-023-42313-3)
Supplement: Supplementary file 1 — Supplementary Information [file 41467_2023_42313_MOESM1_ESM.pdf]

Supplementary Information

Title: Reprogramming of cis-regulatory networks during skeletal muscle atrophy in male mice

Hongchun Lin, Hui Peng*, Yuxiang Sun, Meijun Si, Jiao Wu, Yanlin Wang, Sandhya S Thomas, Zheng Sun, and Zhaoyong Hu*

Assembly of accessible chromatin in mouse skeletal muscles

For snATAC-seq datasets, we aggregated all quality mapped reads from examined animals after batch effect correction (Harmony v1.0) and analyzed several metrics including biological replicates, insert size periodicity, and TSS enrichment. The results were indicative of high-quality snATAC-seq (Supplementary Fig. 1a~c). After quality control, we selected 14,978 normal nuclei plus 11,424 denervated nuclei, from which we obtained 206,818 high-confidence accessible chromatin regions (ACRs or peaks). We then clustered the nuclei based on their ACR similarity using uniform manifold approximation projection (UMAP). This unsupervised analysis revealed 19 major clusters covering all cell types in muscle (Supplementary Fig.1d). To annotate the snATAC clusters, we generated snRNA-seq atlas based on the expression of known cell-type markers (Supplementary Fig.1e~f) and then integrated the snATAC-seq and snRNA-seq datasets using label transfer in Signac [56, 57]. Transfer anchors were identified between the gene activity matrix from the snATAC-seq and snRNA-seq dataset, after which predicted nucleus types were assigned. Subsequently, snATAC clusters were annotated into 11 cell types (Supplementary Fig. 1g), encompassing myonuclei (Type I, Type IIa/x, Type IIb), myotendinous junction nuclei (MTJ), neuromuscular junction nuclei (NMJ), and also nuclei representing satellite cells (MuSCs), FAPs, macrophages, endothelial cells (ECs), pericytes, and adipocytes. To confirm this annotation, we surveyed the chromatin accessibility for marker genes indicative of each cell type in normal muscles and confirmed each of those genes to have a strong peak at the TSS only in the corresponding cell type (Supplementary Fig.1h).

To evaluate the relationship between nuclear transcription and chromatin accessibility, we embedded normal muscle snATAC-seq (n = 14,978) into normal muscle snRNA-seq (n = 15,739). Although both profiles were similar within a given cluster, greater variation was observed in chromatin accessibility (Supplementary Fig.1i), suggesting that chromatin organization and accessibility are more heterogeneous than the transcriptome. This result is expected because ACRs contain both coding genes and their regulatory elements. We then examined the consistency of cluster assignments between snATAC-seq and scRNA-seq data by comparing gene activity scores (4,262 highly variable genes from snATAC clusters) with the

gene expression recorded in snRNA-seq clusters. Pearson's correlation coefficient analysis indicated good concordance in each normal muscle cell type (Supplementary Fig. 1j). We additionally overlapped ACRs from snATAC-seq of normal GAS muscle with a previously published bulk ATAC-seq dataset (GSM3981671) from mouse quadriceps muscle [6]. We found the snATAC-seq data to show good consistency with the bulk dataset; for example, all peaks on chromosome 14 in the bulk dataset were captured by our snATAC-seq (Supplementary Fig. 1k). Notably, although the snATAC results were comparable with the cell type assignments as determined by snRNA-seq, populations having only a small number of nuclei, such as type I and NMJ myonuclei, could not be resolved into individual clusters based on the snATAC-seq dataset (Supplementary Fig.1g). In summary, we constructed cell-type-specific DNA accessibility profiles for mouse skeletal muscle, which can be used to reveal the gene regulatory logic in this tissue.

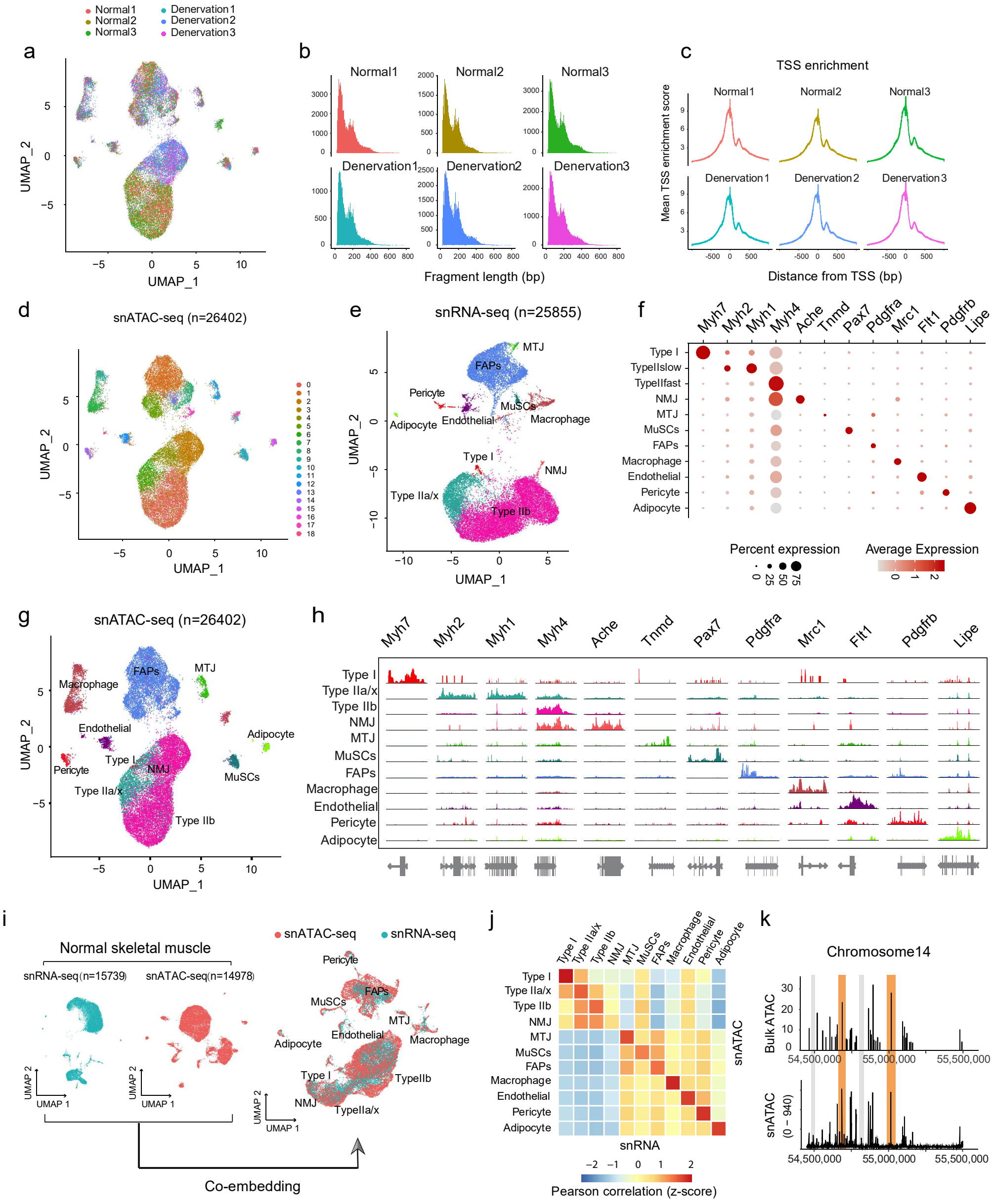

Supplementary Fig. 1: Assembly of accessible chromatin in mouse skeletal muscles.

(a) Biological replicates: UMAP plot displaying snATAC-seq dataset per gastrocnemius muscle sample. Each color represents an independent sample.

(b) Insert size periodicity: insert size distribution of the 6 snATAC-seq samples showing periodic patterns.

(c) TSS enrichment: transcription starts sites (TSS) signal enrichment of the 6 snATAC-seq samples.

(d) UMAP plot displaying the unsupervised clusters of snATAC-seq datasets in Signac. Each cluster is color-coded.

(e) Uniform Manifold Approximation and Projection (UMAP) plot displaying 11 nuclear clusters of snRNA-seq data form normal plus denervated Gas muscle nuclei (n = 25,855), which are colored and labeled according to nuclei identities.

(f) Dot plot represents the expression intensity and density of marker genes within each cell type in the gastrocnemius muscle. Dot size indicates the percentage of nuclei expressing a gene within particular cell types; color intensity indicates the level of average gene expression (“low to high” reflect as gray to red).

(g) UMAP projected snATAC-seq data of normal plus denervated Gas muscle nuclei (n = 26,402) onto two dimensions based on the chromatin accessibility across nuclei. The clusters are colored and labeled according to cell identities.

(h) CoveragePlot visualizing the read density in each snATAC-seq cluster at the transcription start sites (TSS) of cell-type marker gene.

(i) UMAP plots showing the co-embedding of normal snRNA-seq (blue) and snATAC-seq (pink) dataset.

(j) Heatmap showing Pearson’s correlation coefficients between gene activity (snATAC-seq) and gene expression (snRNA-seq) in normal GAS muscle.

(k) Fragment coverage plot displaying peak regions over chromosome 14 in aggravated snATAC-seq and bulk ATAC-seq data. Example peaks that are identified in both datasets are colored orange; the dataset-specific peaks are gray.

Source data are provided as a Source Data file.

Supplementary Figure 2.

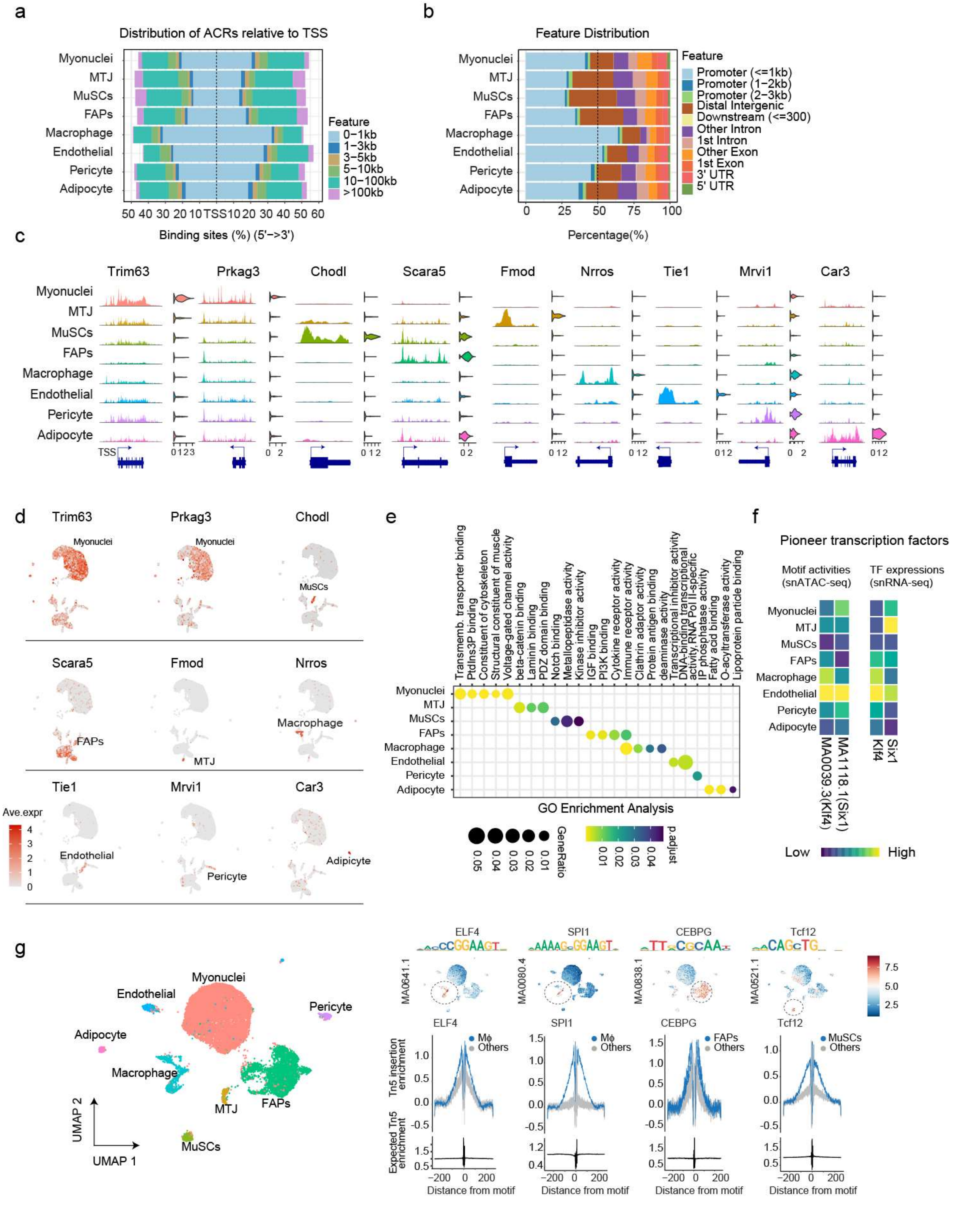

Supplementary Fig. 2. Cell type specific ACRs and their corresponding function in normal muscle.

(a) Relative distance of ACRs to TSS in each cell type.

(b)Bar plot of annotated ACRs locations for each cell type.

(c) Fragment coverage around the promoter of ACRs in different nucleus types (Left). Violin plots showing average gene activities of specific genes in each nucleus types (Right).

(d) UMAP plot displaying the RNA expression of nucleus-type specific ACRs related genes in snRNA-seq data. The color scale represents the relative level of gene expression: grey indicates low level of and red indicates high levels of expression.

(e) Dot pot comparison of GO function enrichment results among nucleus-type specific ACRs. Dot color represents p-value (Benjamini-Hochberg-adjusted one-sided hypergeometric test), dot size represents the gene ratio of a GO term.

(f) Z-score heatmaps showing two pioneer TFs' motif activity (left) and the corresponding TF gene expression (right) for each nuclei type.

(g) UMAP plots displaying the nuclei type in normal GAS muscle (left) and displaying additional 4 TFs' motif activities and their footprinting plots, footprinting lines were colored by cell identities (right).

Source data are provided as a Source Data file.

Supplementary Figure 3

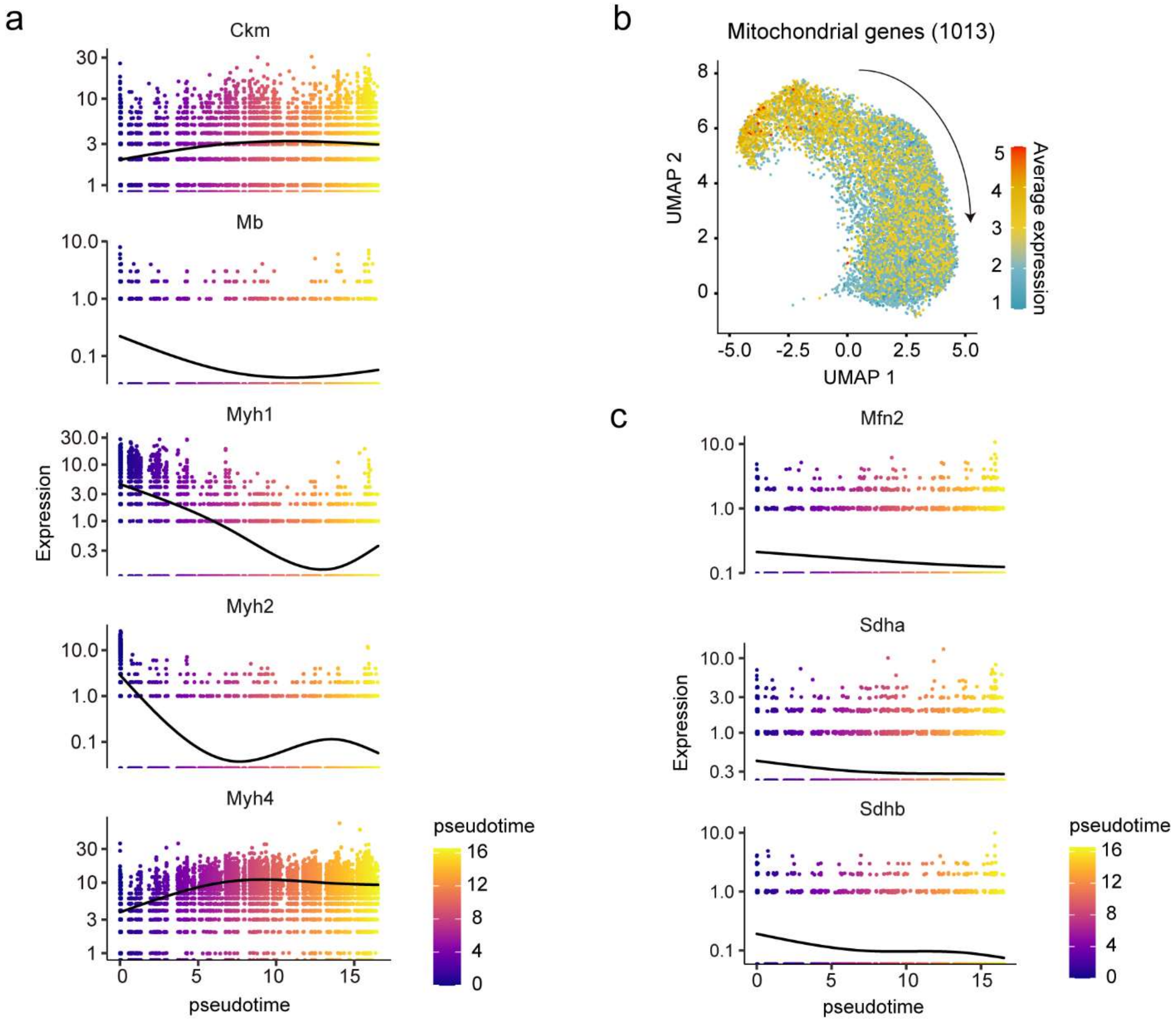

Supplementary Fig. 3. Dynamic chromatin accessibility and transcriptome in myonuclear transition.

(a) Gene expression dynamics of *Ckm*, *Mb*, *Myh1*, *Myh2* and Myh4 gene along the pseudotime trajectory of myonuclei.

(b) UMAP plots showing average expression of 1013 mitochondrial genes in normal myonuclei.

(c) Gene expression dynamics of selected mitochondrial gene along the pseudotime trajectory of myonuclei.

Supplementary Figure 4

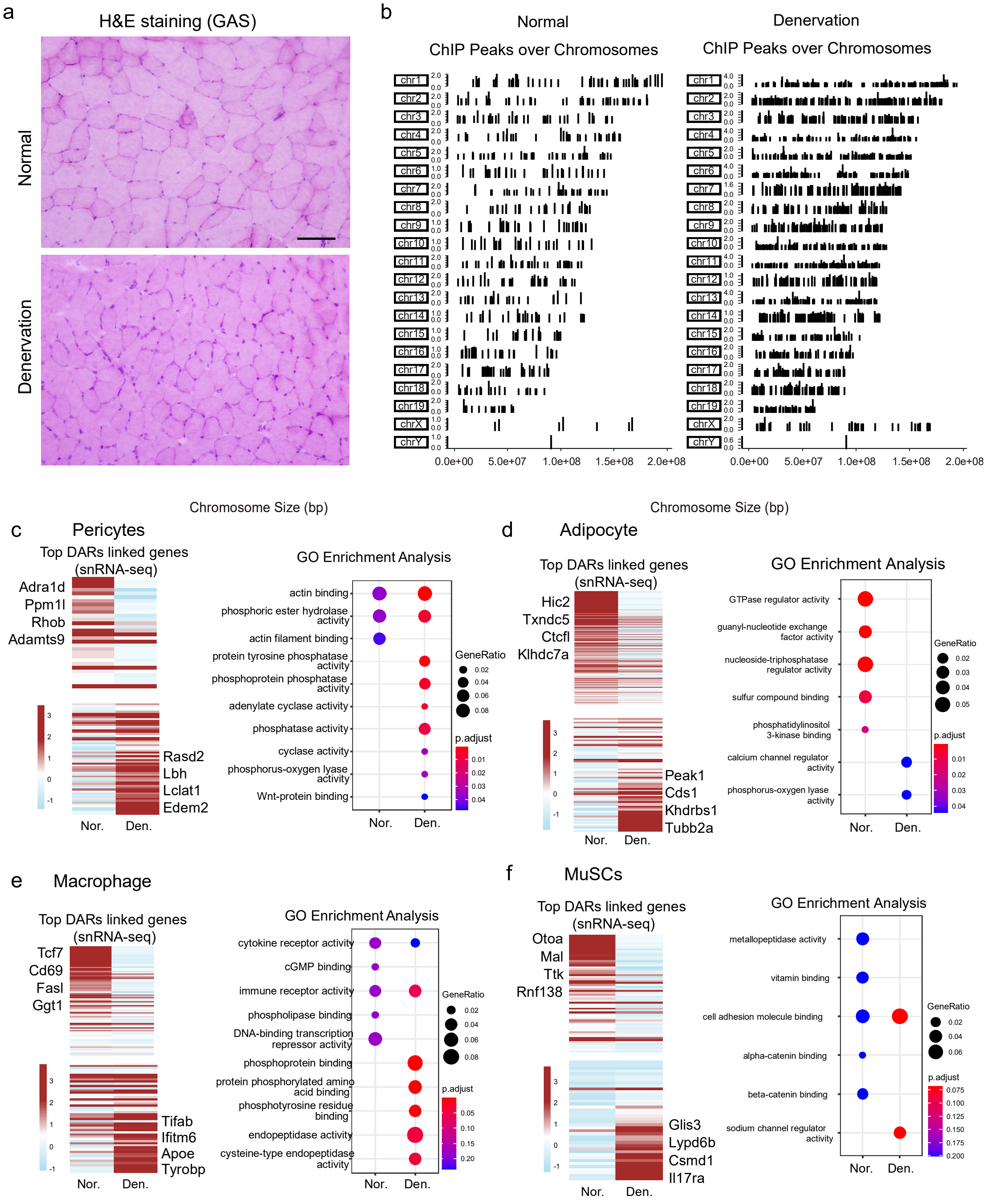

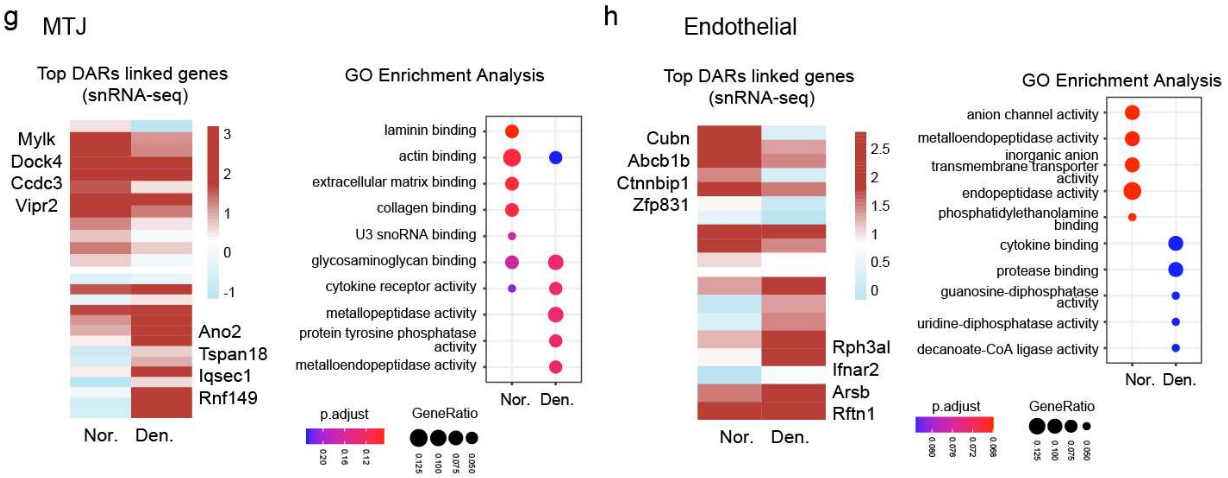

Supplementary Fig. 4 Gene expression of cell type specific DARs and their corresponding GO function in denervation comparing to normal in other muscle resident cells.

(a) H&E staining of normal (upper) and denervated (lower) GAS. Scale bar: 50μm.

(b) Fragment coverage plot displaying peak regions over 21 chromosomes in normal and denervation muscle in snATAC-seq data.

(c-h) Heatmaps (left) and dot plots (right) displaying expression levels of genes that were linked by co-accessibility to DARs and their GO functions in pericytes (c), adipocyte (d), macrophages (e), MuSCs (f), MTJ (g) and endothelial cells (h). P values are calculated using Benjamini-Hochberg-adjusted one-sided hypergeometric test. Nor: Normal, Den: Denervation.

Supplementary Figure 5

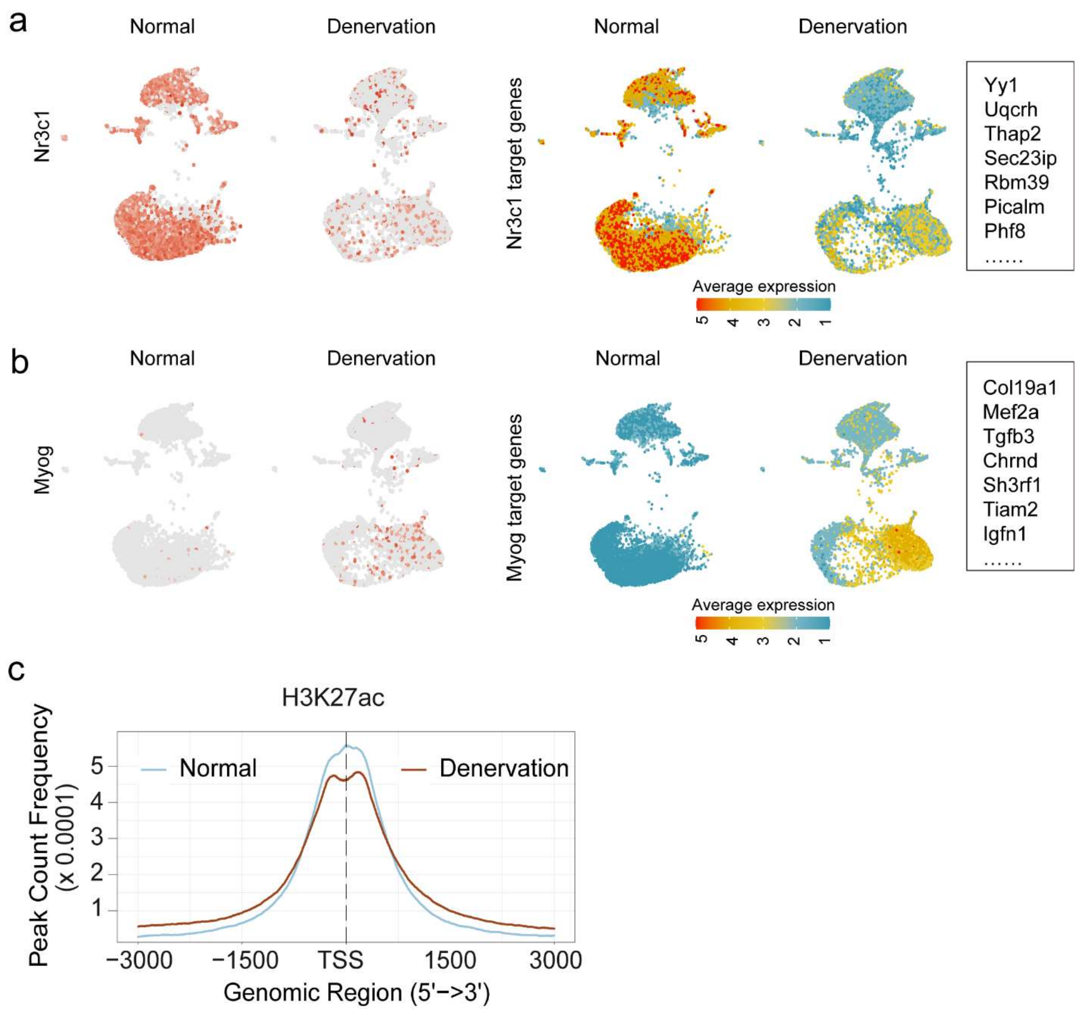

Supplementary Fig. 5 Cell-type specific changes of cis-regulation in denervated muscle

(a) UMAP plots showing the down-regulation of *Nr3c1* plus its target genes in response to denervation.

(b) UMAP plots showing the up-regulation of *Myog* plus its target genes in response to denervation.

(c) H3K27ac ChIP-seq profile of normal and denervated muscles: the read count frequency in selected range around TSS (<3000bp).

Supplementary Figure 6

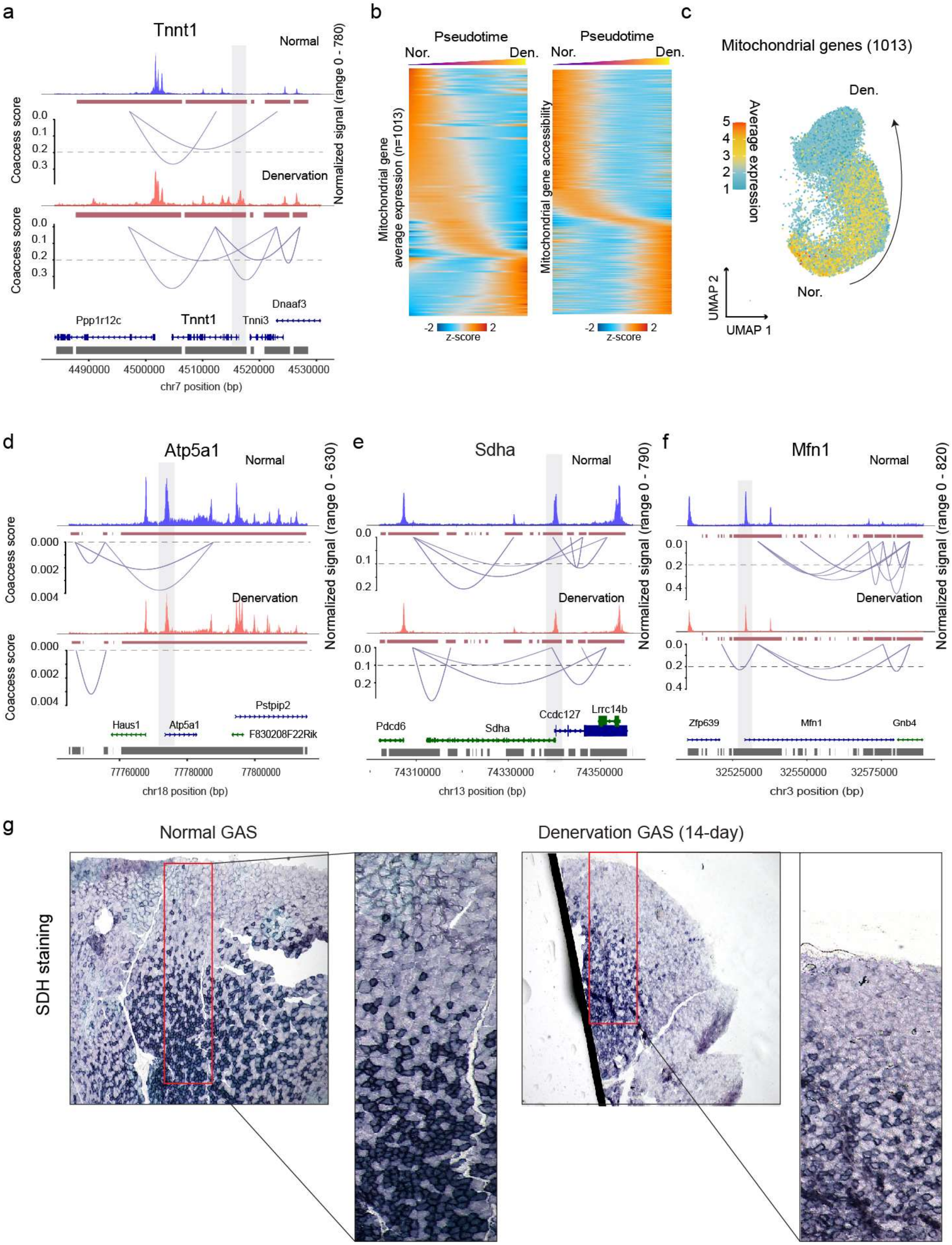

Supplementary Fig. 6 Decreasing of mitochondrial gene accessible and cis-regulated networks in denervated myonuclei.

(a) Fragment coverage tracks showing chromatin accessibility in normal and denervated myonuclei. Purple lines indicate cis-coaccessibility networks around *Tnnt1* in normal and denervated myonuclei. Grey shaded areas, Tnnt1 promoter regions that had increased accessibility in denervated muscle.

(b) Heatmap displaying average expression of 1016 mitochondrial gene (left) and their corresponding gene accessibility (right) over pseudotime trajectory across scRNA-seq and snATAC-seq myonuclei. The color scale represents a z-score scaled by row.

(c) UMAP plots showing average expression of 1013 mitochondrial genes in normal myonuclei. Nor: Normal, Den: Denervation

(d-f) Fragment coverage tracks showing chromatin accessibility in normal and denervated myonuclei. Purple lines indicate cis regulatory networks around mitochondrial gene *Atp5a1* (d), *Sdha* (E) and *Mfn1* (f) in normal and denervated myonuclei. Grey shaded areas, promoter regions that had decreased accessibility in denervated muscle.

(g) Succinate dehydrogenase (SDH) staining demonstrating oxidative capacity in normal (left 40X， right 200X) and denervated (14 days) TA muscles.

Supplementary Figure 7

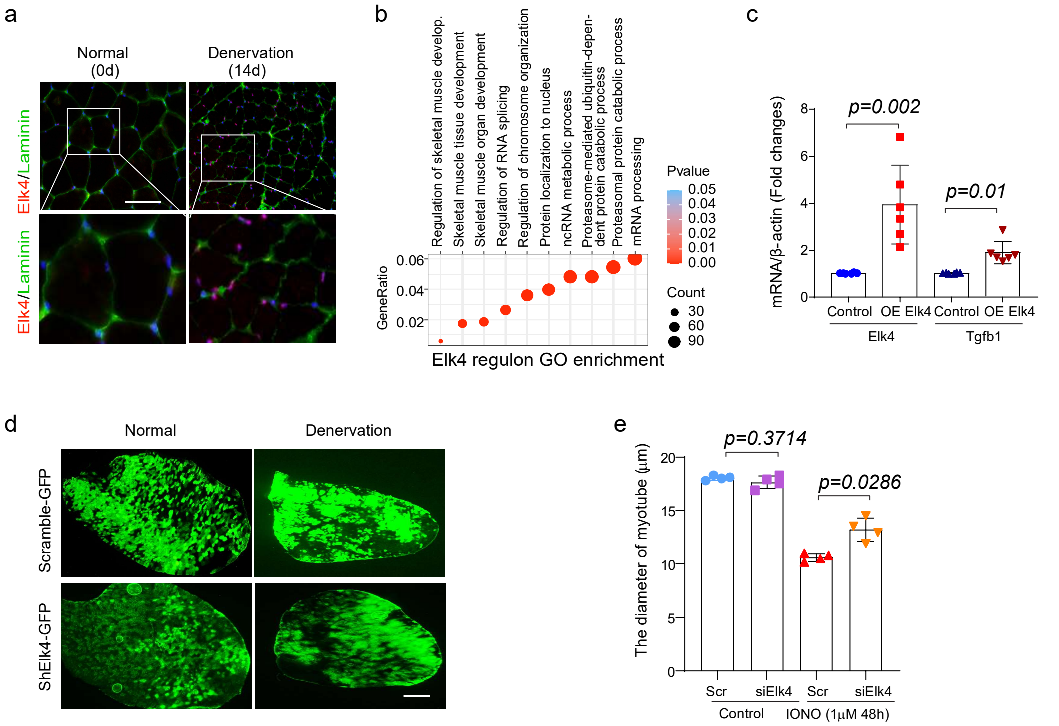

Supplementary Fig. 7 Elk4 regulated muscle atrophy.

(a) Immunofluorescence staining of *Elk4* in normal (0 days) and denervated (14 days) Gas muscle. Red: Elk4; green: Laminin; blue DAPI. Scale bar: 50 μm.

(b) GO process enrichment of *Elk4* target genes. Dot color represents p-value (Benjamini-Hochberg-adjusted one-sided hypergeometric test), dot size represents the gene number that enrichment in a GO term.

(c) Quantitative RT-PCR analysis of *Elk4* and *Tgfb1* mRNA in control group and *Elk4* overexpression group of mice C2C12 cell. Fold change represents the relative expression of the mRNA in comparison to the control group. β-actin serves as the loading control. Data shown as mean ± SEM from 6 experimental repeats. Significance was assessed using a two-side Wilcoxon rank-sum test.

(d) Cross-sections fluorescence images evaluating the transfection efficiency of TA muscles from experimental mice. Scale bar: 200 μm.

(e) The calculation of diameter of myotubes in Fig.6k. Data shown as mean ± SEM from 4 experimental repeats. Significance was assessed using two-side one-way ANOVA with post hoc Tamhane's multiple comparisons test.

Source data are provided as a Source Data file.
