# Supplementary material for: Reprogramming of cis-regulatory networks during skeletal muscle atrophy in male mice
Source: Nat Commun. 2023 Oct 18;14:6581. doi: 10.1038/s41467-023-42313-3 (PMC10584982; doi:10.1038/s41467-023-42313-3)
Supplement: Supplementary file 3 — Reporting Summary [file 41467_2023_42313_MOESM3_ESM.pdf]

nature portfolio

Corresponding author(s): Zhaoyong Hu
Last updated by author(s): Sep 6, 2023

Reporting Summary

Nature Portfolio wishes to improve the reproducibility of the work that we publish. This form provides structure for consistency and transparency in reporting. For further information on Nature Portfolio policies, see our Editorial Policies and the Editorial Policy Checklist.

Statistics

For all statistical analyses, confirm that the following items are present in the figure legend, table legend, main text, or Methods section.

n/a	Confirmed	
☐	☒	The exact sample size (*n*) for each experimental group/condition, given as a discrete number and unit of measurement
☐	☒	A statement on whether measurements were taken from distinct samples or whether the same sample was measured repeatedly
☐	☒	The statistical test(s) used AND whether they are one- or two-sided<br>*Only common tests should be described solely by name; describe more complex techniques in the Methods section.*
☐	☒	A description of all covariates tested
☐	☒	A description of any assumptions or corrections, such as tests of normality and adjustment for multiple comparisons
☐	☒	A full description of the statistical parameters including central tendency (e.g. means) or other basic estimates (e.g. regression coefficient) AND variation (e.g. standard deviation) or associated estimates of uncertainty (e.g. confidence intervals)
☐	☒	For null hypothesis testing, the test statistic (e.g. *F*, *t*, *r*) with confidence intervals, effect sizes, degrees of freedom and *P* value noted<br>*Give P values as exact values whenever suitable.*
☐	☒	For Bayesian analysis, information on the choice of priors and Markov chain Monte Carlo settings
☐	☒	For hierarchical and complex designs, identification of the appropriate level for tests and full reporting of outcomes
☒	☐	Estimates of effect sizes (e.g. Cohen's *d*, Pearson's *r*), indicating how they were calculated

Our web collection on statistics for biologists contains articles on many of the points above.

Software and code

Policy information about availability of computer code

Data collection	Single nucleus RNA and ATAC datasets are aligned and counted with cellranger v3.0 and cellranger-atac v2.0
Data analysis	Single cell RNA and atac seq datasets were analyzed in R 4.1.0 using Seurat v 4.1.4, Signac V4.0, Monocle V3, chromVAR V1.14.0 , Cicero V1.3.8, clusterProfiler V4.0.5 , ChIPseeker 1.26.2, circlize V0.4.15. H3K27ac Chip-seq dataset was analysed with Cutadapt 1.9 , Trimmomatic v0.38 ,Bowtie2 , samtools 1.9. RT-qPCR and western blot quantification analysis were performed by GraphPad prism7.0 or SPSS26.0.

For manuscripts utilizing custom algorithms or software that are central to the research but not yet described in published literature, software must be made available to editors and reviewers. We strongly encourage code deposition in a community repository (e.g. GitHub). See the Nature Portfolio guidelines for submitting code & software for further information.

Data

Policy information about availability of data

All manuscripts must include a data availability statement. This statement should provide the following information, where applicable:

- Accession codes, unique identifiers, or web links for publicly available datasets
- A description of any restrictions on data availability
- For clinical datasets or third party data, please ensure that the statement adheres to our policy

All of the data for this manuscript have been made publicly-available. The accession number for the sequencing data reported in this paper is NCBI GEO: GSE183802 (snRNA-seq), GSE217576 and GSE217577 (bulk RNA-seq and snATAC-seq, H3K27ac-ChIP-seq).

Research involving human participants, their data, or biological material

Policy information about studies with human participants or human data. See also policy information about sex, gender (identity/presentation), and sexual orientation and race, ethnicity and racism.

Reporting on sex and gender	N/A.
Reporting on race, ethnicity, or other socially relevant groupings	N/A
Population characteristics	N/A
Recruitment	N/A
Ethics oversight	N/A

Note that full information on the approval of the study protocol must also be provided in the manuscript.

Field-specific reporting

Please select the one below that is the best fit for your research. If you are not sure, read the appropriate sections before making your selection.

☒ Life sciences ☐ Behavioural & social sciences ☐ Ecological, evolutionary & environmental sciences

For a reference copy of the document with all sections, see nature.com/documents/nr-reporting-summary-flat.pdf

Life sciences study design

All studies must disclose on these points even when the disclosure is negative.

Sample size	Sample sizes for each experiment are indicated in the figure legends. We did not use a statistical method to predetermine sample size. The number of animals or cells for the experiments was determined based on our experience and prior studies in this field.
Data exclusions	In both snATAC-seq and snRNA-seq analyses, low-quality nuclei were excluded from the aggregated datasets according to the analysis process protocol in 10X Genomic. For further details, please refer to the relevant section in the Method.
Replication	All experiments were taken at lease 4 times (biological replicates) with similar results.
Randomization	Age and body weight matched male mice was randomly separated into different experimental groups.
Blinding	The investigators were not blinded during the experiments or outcome assessment because the atrophy phenotype can be readily identified by visual inspection.

Reporting for specific materials, systems and methods

We require information from authors about some types of materials, experimental systems and methods used in many studies. Here, indicate whether each material, system or method listed is relevant to your study. If you are not sure if a list item applies to your research, read the appropriate section before selecting a response.

Materials & experimental systems

n/a	Involved in the study	
☐	☒	Antibodies
☐	☒	Eukaryotic cell lines
☒	☐	Palaeontology and archaeology
☐	☒	Animals and other organisms
☒	☐	Clinical data
☒	☐	Dual use research of concern
☒	☐	Plants

Methods

n/a	Involved in the study	
☐	☒	ChIP-seq
☒	☐	Flow cytometry
☒	☐	MRI-based neuroimaging

Antibodies

Antibodies used	Primary antibodies:<br>1.Elk4 antibody, Novus Biologicals, Cat # NBP1-87092

2.Phospho-Smad3 (Ser423/425) antibody, Cell Signaling Technology, Cat # 9520 (RRID:AB_2193207)
3.Tgfb1 antibody, Sigma, Cat # SAB5300197
4.β-actin antibody, Santa cruz, Cat # sc47778 (RRID:AB_626632)
5.Acetyl-Histone H3 (Lys27) (D5E4) XP® Rabbit mAb, Cell Signaling Technology, Cat # 8173 (RRID:AB_2798746)
6.DAP Kinase 1 (DAPK1) Rabbit pAb, ABclonal, Cat # A5741
7.RPS6KC1 Rabbit pAb, ABclonal, Cat # A13807
8.TRIM63 Rabbit pAb, ABclonal, Cat # A3101
9.CHODL Rabbit pAb, ABclonal, Cat # A17827
10.PRKAG3 Rabbit pAb, ABclonal, Cat # A14132
11.LDLRAD3 Antibody, Novus Biologicals, Cat # NBP1-86261
12.SCARA5 Antibody, Novus Biologicals, Cat # NBP1-83572
13.Myh2 Antibody DSHB Cat# 2F7 (RRID: AB_1157865)
14.Myh4 Antibody DSHB Cat# BF-F3 (RRID:AB_2266724)
15.Myh7 Antibody DSHB Cat# BA-D5 (RRID: AB_2235587)
16.Pax7 Antibody DSHB Cat#PAX7 (RRIDAB_528428)
Secondary antibodies :
1.Donkey Anti-Mouse (Invitrogen; A21202 or A21203)
2.Donkey Anti-Rabbit (Invitrogen; A21206 or A21207)
3.Goat anti-Rabbit (Invitrogen;HRP,31460)
4.Goat anti-Mouse (Invitrogen;HRP,G21040)

Validation

The antibodies were validated by manufacturer as below:
Antigen species application Link
1.Elk4; Rabbit; WB, ICC/IF, IHC; https://www.novusbio.com/products/elk4-antibody_nbp1-87092
2.Phospho-Smad3 (Ser423/425);Rabbit; WB,IP;https://www.cellsignal.com/products/primary-antibodies/phospho-smad3-ser423-425-c25a9-rabbit-mab/9520?site-search-type=Products&N=4294956287&Ntt=phospho-smad3+%28ser423%2F425%29+%28c25a9%29+rabbit+mab&fromPage=plp&_requestid=577521&country=USA&_requestid=576129
3.Tgfb1; mouse;WB, IHC; https://www.sigmaaldrich.cn/CN/zh/product/sigma/sab5300197
4.Beta-actin,Rabbit, WB; https://www.scbt.com/p/beta-actin-antibody-c4?requestFrom=search
5.H3k27ac; Rabbit; WB,IF,F,ChIP; https://www.cellsignal.com/products/primary-antibodies/acetyl-histone-h3-lys27-d5e4-xp-rabbit-mab/8173
6.DAPK1; Rabbit; WB,IHC-P; https://abclonal.com.cn/catalog/A5741
7.RPS6KC1; Rabbit; WB; https://abclonal.com.cn/catalog/A13807
8.TRIM63; Rabbit; WB,IF,ICC,IHC-P; https://abclonal.com.cn/catalog/A3101
9.CHODL; Rabbit; WB; https://abclonal.com.cn/catalog/A17827
10.PRKAG3; Rabbit; WB,IF/ICC; https://abclonal.com.cn/catalog/A14132
11.LDLRAD3; Rabbit; WB, ICC/IF, IHC; https://www.novusbio.com/products/ldlrad3-antibody_nbp1-86261
12.SCARA5; Rabbit; IHC; https://www.novusbio.com/products/scara5-antibody_nbp1-83572
13.MYH2; Mouse; WB,IF,IHC; https://dshb.biology.uiowa.edu/2F7
14.MYH4; Mouse; WB,IF,IHC,ELISA; https://dshb.biology.uiowa.edu/BF-F3
15.MYH7; Mouse; IF,IHC,WB; https://dshb.biology.uiowa.edu/BA-D5
16.PAX7; Mouse; WB,IF,IHC,IP,FACS,FFPE; https://dshb.biology.uiowa.edu/PAX7

Eukaryotic cell lines

Policy information about cell lines and Sex and Gender in Research

Cell line source(s)	Mouse C2C12 (CRL-1772) from American Type Culture Collection.
Authentication	C2C12 cell line were authenticated by expression of expected skeletal muscle markers such as MyoD and MYH.
Mycoplasma contamination	C2C12 tested negative for myocoplasma contamination.
Commonly misidentified lines (See ICLAC register)	Not any.

Animals and other research organisms

Policy information about studies involving animals; ARRIVE guidelines recommended for reporting animal research, and Sex and Gender in Research

Laboratory animals	Male C57BL/6J (12-week-old) mice were fed ad libitum on a standard laboratory diet, maintained under a 12-h light/dark cycle conditions.
Wild animals	NO
Reporting on sex	Only adult male mice were used in this study. We aimed at investigating the function role of Elk4 in normal and denervated mice. We have indicated information of gender in the title and abstract section of our revised manuscript.
Field-collected samples	No field collected samples were used in the study.
Ethics oversight	Animal procedures were approved by the Baylor College of Medicine Institutional Animal Care and Use Committee or by the Ethics Committee of the Third Affiliated Hospital of Sun Yat-sen University.

Note that full information on the approval of the study protocol must also be provided in the manuscript.

Plants

Seed stocks	*Report on the source of all seed stocks or other plant material used. If applicable, state the seed stock centre and catalogue number. If plant specimens were collected from the field, describe the collection location, date and sampling procedures.*
Novel plant genotypes	*Describe the methods by which all novel plant genotypes were produced. This includes those generated by transgenic approaches, gene editing, chemical/radiation-based mutagenesis and hybridization. For transgenic lines, describe the transformation method, the number of independent lines analyzed and the generation upon which experiments were performed. For gene-edited lines, describe the editor used, the endogenous sequence targeted for editing, the targeting guide RNA sequence (if applicable) and how the editor was applied.*
Authentication	*Describe any authentication procedures for each seed stock used or novel genotype generated. Describe any experiments used to assess the effect of a mutation and, where applicable, how potential secondary effects (e.g. second site T-DNA insertions, mosiacism, off-target gene editing) were examined.*

ChIP-seq

Data deposition

☒ Confirm that both raw and final processed data have been deposited in a public database such as GEO.

☐ Confirm that you have deposited or provided access to graph files (e.g. BED files) for the called peaks.

Data access links<br>*May remain private before publication.*	The accession number for the sequencing data reported in this paper is NCBI GEO: GSE217577 (H3K27ac-ChIP-seq).
Files in database submission	Files are deposit in https://www.ncbi.nlm.nih.gov/geo/query/acc.cgi.
Genome browser session<br>(e.g. UCSC)	UCSC

Methodology

Replicates	Two replicates of mice muscles in normal and denervated group were performed.
Sequencing depth	Average total raw reads: 66989992.<br>Average uniquely Mapped Reads:49588353.<br>Sequencing depth: 50 millions.
Antibodies	Acetyl-Histone H3 (Lys27) (D5E4) XP® Rabbit mAb
Peak calling parameters	The Clean data were quality controlled with FastQC. Clean reads were aligned to the mouse reference genome (mm10) by bowtie2 (v2.3.5.1). The mapping data were analyzed with the MACS2 (v.2.2.7.1) peak-calling algorithm. High-confidence peak screening between samples using the IDR program.
Data quality	High-quality mapped reads (MPAQ greater than or equal to 30) were used for subsequent information analysis.
Software	FastQC.https://www.bioinformatics.babraham.ac.uk/projects/fastqc/<br>MACS2 (v.2.2.7.1)https://hbctraining.github.io/Intro-to-ChIPseq/lessons/05_peak_calling_macs.html<br>Cutadapt Martin.M (2017) https://github.com/marcelm/cutadapt<br>Trimmomatic Bolger, A. M.(2014) USADELLAB.org - Trimmomatic: A flexible read trimming tool for Illumina NGS data<br>Fastqc Babraham Institute Babraham Bioinformatics - FastQC A Quality Control tool for High Throughput Sequence Data<br>Bowtie2 Langmead B.et.al.(2012) https://bowtie-bio.sourceforge.net/bowtie2/manual.shtml<br>samtools Danecek.P.et.al.(2021) https://github.com/samtools/samtools<br>clusterProfiler V4.0.5 Yu G et al. (2015) https://github.com/YuLab-SMU/clusterProfiler<br>ChIPseeker Yu G et al. (2015) https://bioconductor.org/packages/release/bioc/vignettes/ChIPseeker/inst/doc/ChIPseeker.html
