# Supplementary material for: Reprogramming of cis-regulatory networks during skeletal muscle atrophy in male mice
Source: Nat Commun. 2023 Oct 18;14:6581. doi: 10.1038/s41467-023-42313-3 (PMC10584982; doi:10.1038/s41467-023-42313-3)
Supplement: Supplementary file 4 — Source Data [file 41467_2023_42313_MOESM4_ESM.zip › Description of Source Data Files.docx]

**Description of Source Data Files**

**Source Data**

Source Data files correspond to all data presented in the graphs of all figures. Additionally, uncropped scans of all blots and gels for each figure are provided.

**Supplementary Data Legends**

Supplementary Data 1: DARs among distinct cell types in the skeletal muscle of a normal mouse, related to Fig.1d.

Supplementary Data 2: List of motifs and TFs in normal muscle, related to Fig.1f.

Supplementary Data 3: All DAR-associated genes in both normal and denervated muscles, related to Fig.3f.

Supplementary Data 4: List of 579 motifs with their best-matched TFs, related to Fig.4a.

Supplementary Data 5: List of 125 mutual TFs, related to Fig.4c.

Supplementary Data 6: List of candidate enhancers with increased accessibility in denervated muscle, related to Fig.4f.

Supplementary Data 7: List of candidate enhancers with decreased accessibility in denervated muscle, related to Fig.4f.

Supplementary Data 8: TF hierarchy and GO of target genes, related to Fig.5g.
